# Annual Solar Cycles: Investigating Seasonal Variations in Southern Norway and Their Potential Role in Human Eye Growth

**DOI:** 10.1167/iovs.67.10.54

**Published:** 2026-08-21

**Authors:** Rigmor C. Baraas, Stuart J. Gilson

**Affiliations:** 1 National Centre for Optics, Vision and Eye Care, University of South-Eastern Norway, Kongsberg, Norway

**Keywords:** eye growth, daylight, emmetropization, opsin-weighted illuminance

## Abstract

**Purpose:**

Human eye growth is reported to follow a seasonal pattern, with accelerated eye growth leading to myopia being linked to limited exposure to daylight. To investigate how environmental lighting may contribute to eye growth and refractive errors in emmetropic children, we analyzed real-world seasonal variations in daylight intensity, duration, and spectrum and their association with healthy regulated human eye growth.

**Methods:**

Downwelling irradiance was collected around the 2 solstice and the 2 equinox dates to assess variations across 4 seasons at 60°N latitude location over 4 years (2022–2025). To assess seasonal changes in the relative levels of short- versus long-wavelength parts of daylight, we calculated ratios of alpha-opic equivalent daylight illuminance (EDI) for the photoreceptors. Gold-standard ocular measurements were obtained from emmetropic 7 to 11-year-old children who spend 1 to 2 hours outdoors every day and were used to explore the associations between healthy eye growth rates and seasonal variations in these ratios.

**Results:**

Daylight intensities were significantly lower during autumn and winter mornings, winter midday, as well as autumn and spring afternoons. The spectra were significantly short-wavelength enriched during winter mornings and midday, and spring afternoons. The seasonal cycle of daylight and human eye growth in 7 to 11-year-old children was in anti-phase, with peak growth delayed relative to the shortest winter days when daylight was short-wavelength enriched.

**Conclusions:**

The short-wavelength enrichment encompasses the sensitivities of both S opsin and melanopsin and invites speculation that the eye may have evolved to exploit seasonal variations in intensity and spectrum as cues that may contribute to the regulation of eye growth via photoreceptor opsin activation.

The Earth's axial tilt induces variations in intensity and spectral composition of the solar energy that reaches its surface, as it orbits the sun (annual solar cycle). The degree of variability depends on the observer's geographic position on the Earth, the time of year, prevailing weather conditions, and atmospheric composition.^1^ Daylight, a fundamental component of the outdoor visual environment, provides essential illumination and is argued to be biologically necessary for general human health.^2^ It plays a key role in regulating circadian rhythms^3^ and may also contribute to the regulation of eye growth.^4^ Increased outdoor exposure has been associated with a reduced risk of myopia (near-sightedness) even when children have high levels of near work.^5^ Myopia is increasingly prevalent, particularly in East Asian countries^6^ where its rapid rise is often attributed to lifestyle factors, such as limited outdoor activity.^5^ Myopia typically results from excessive ocular axial elongation, a disruption of the eye’s ability to regulate growth to achieve emmetropia (matching eye length to optical power ). Indoor artificial light environments are both of low intensity and deficient in blue (short wavelengths) is used in support of the myopia epidemic in countries where children spend very little time outdoors during the day, and where the culture is not encouraging outdoor time during recess.^7^ Clinical trials where outdoor time has been mandated or otherwise promoted have shown noticeable decreases in both the incidence and prevalence of myopia.^8^^,^^9^ Experimental studies on young adults have implicated that exposure to narrow-band blue light for 1 hour has the effect of inhibiting unwanted eye growth.^10^

Exposure to bright daylight is hypothesized to contribute to the regulation of eye growth by increasing the release of retinal dopamine (DA).^5^ Findings from animal studies have refined this hypothesis, suggesting that light-induced DA release may act as a regulatory brake mechanism, with high-intensity light inhibiting excessive axial eye growth.^11^ Conversely, artificially-enforced constant darkness or constant light and the associated lack of diurnal changes in exposure to light/dark, appears to dysregulate the eye’s growth mechanism,^12^ suggesting that circadian entrainment influences eye growth regulation (for a review, see Chakraborty et al., 2018^13^). A complementary theory proposes that the light's spectrum is needed for eye-growth regulation and that blue-rich daylight enables the eye to assess a “signed defocus signal” to determine whether the eye is too short (hyperopic) or too long (myopic).^14^ This feedback-loop is achieved by evaluating which wavelengths of light are better focussed, based on the eye's longitudinal chromatic aberration (LCA), whereby shorter (bluer) wavelengths focus closer to the front of the eye compared to longer (redder) wavelengths.^15^

Together, these hypotheses suggest that natural daylight is important for regulating eye growth by delivering three components: (1) high-intensity light to drive DA release in the retina, (2) diurnal variation in light exposure to drive circadian entrainment, and (3) a broad-band white light with balanced spectral composition that allows for detection of defocus as described above. It is uncertain, however, if spectral composition plays a determining role in regulating eye growth.^4^ This is of particular interest under conditions such as the dim light of winter, a time of year that is associated with increased eye growth both in emmetropes and myopes.^16^^–^^18^ We raise the question of whether there is a difference in relative levels of short- (blue) versus long- (red) wavelength light during the dim conditions of winter compared with bright conditions of summer, and to what degree such a difference can be used to infer if the eye may use spectral composition in tandem with intensity for regulating eye growth. If dim light is important for promoting eye growth, will growth be regulated if the light is rich in blue, but dysregulated (grows too much) if the light is blue deficient?

Interestingly, children who grow up in Norway and other Nordic countries appear to be at somewhat lower risk of myopia, in that the prevalence is low and stable despite increasing time spent in education and indoors.^19^^–^^22^ The Nordics experience significant seasonal variations in daylight. In South-eastern Norway, at latitude 60°N, it has been reported that children who maintain emmetropia (or low hyperopia) experience regulated eye growth during winter, but during summer eye growth almost ceases.^18^ At this latitude, there is nearly continuous daylight during the summer, whereas winter (November through January) provides merely 6 to 8 hours of daylight. At this latitude, the dominance of blue light (“blåtimen” or “blue hour”) is distinctly perceptible during clear winter mornings and afternoons.

To evaluate the extent to which daylight intensity and spectral composition vary across seasons and to infer if the eye may use this information (either in tandem or independently) to regulate eye growth, we measured and analyzed seasonal variations in daylight at latitude 60°N. In this context, we (1) investigated temporal changes in daylight at the solstices and equinoxes over a 4-year period, using downwelling irradiance data collected from 9-day intervals centered at each equinox/solstice annually, and analyzed variations in both intensity and spectral composition. Within the framework of a 10-year-old child's daily outdoor exposure and the spectral transmittance properties of their eyes, we characterized the seasonal variations in (2) average, and (3) cumulative available illuminance across different time intervals of the day when children are expected to be outdoors. We (4) predicted seasonal changes in the relative levels of short- versus long-wavelength parts of daylight by estimating ratios of average and cumulative available alpha-opic illuminance among the three canonical photoreceptors and intrinsically photosensitive retinal ganglion cells (ipRGCs). Finally, we (5) assessed the relationship between seasonal variation in eye growth and the relative ratios of short- to long-wavelength components of daylight.

## Methods

### Daylight Measurements

Measurements of the sky's spectral power distribution were performed on the roof of the main building at the university's campus in the city of Kongsberg, Norway (59.6647°N−9.6440°E; ground elevation above sea level: 161 m, height of building: 22.3 m, sensor mount: 1.3 m). There were no shadow-casting structures (roof-top furniture, trees, buildings, and pylons) within 0.5 km of this location, ensuring uninterrupted visibility of the sky except for the hills defining the edges of the valley in which Kongsberg lies. Downwelling absolute irradiance was collected from a 180-degree field-of-view CC-3-UV-S Cosine Corrector (Ocean Optics, Inc., Dunedin, FL, USA) optical diffuser (diameter 3.9 mm) coupled to an Ocean Optics HR4000CG-UV-NIR Composite-grating Spectrometer with a single-strand optical fiber. This is a high-resolution spectrometer with spectral resolution of ≈0.257 nm. Measurements were obtained using OceanView (version 1.6.5, Ocean Optics Inc.) software at every minute of every day (1440 measurements per day) for every day of the year, for the wavelength range of 350 to 1130 nm. Thus, it included the longer wavelengths of UV-A (350–400 nm), visible light (400–780 nm), and the near infrared (780–900 nm).

### Calibration, Quality Control, and Filtering of Data

The spectrometer was controlled by the supplied OceanView software, running on a dedicated Apple MacBook Pro. An absolute irradiance template project, packaged with OceanView, was configured and the built-in calibration procedure was followed, which included a dark calibration (capturing a representation of the electrical noise in the system) and a light calibration (against a known light source [halogen lamp, HL-2000-CAL, Ocean Optics Inc.]). The spectrometer was configured to record a spectrum as the average of 5 contiguous measurements each with a 2-second integration time, chosen to avoid saturation on bright days but at the expense of reduced sensitivity in low-light conditions. The spectrometer's electric dark mode and nonlinearity correction were enabled, as per Ocean Optics recommendations, and boxcar averaging (window width = 5) was used to smooth the spectrum before being stored in a database. All spectra were indexed by local time (nominally UTC+1 but advancing to UTC+2 during Daylight Saving Time). The system was re-calibrated yearly, or whenever the cosine corrector was replaced due to damage.

All absolute irradiance measurements included here were downsampled to the 360 to 830 nm range in 1-nm increments for subsequent processing. Due to undetected calibration errors or cosine corrector damage/contamination, some spectra contained anomalously high intensities at wavelengths 363 to 372 nm and 576 to 581 nm. A linear interpolation of intensities was applied over these wavelengths for all spectra to ensure these measurement errors did not unduly influence subsequent analysis.

To document temporal changes in daylight that are considered to have implications for human health, we chose to calculate and analyze photopic illuminance and alpha-opic equivalent daylight illuminance (EDI), rather than what is commonly referred to as photosynthetically active radiation (spectrally integrated visible radiation in the range 400–700 nm).

### Generation of Age-Specific Photoreceptor Fundamentals

Stockman and Rider, 2023,^23^ published functions for generating L-, M-, and S-cone opsin fundamentals which mimic the shape of the CIE 2006 cone sensitivities to an acceptably high degree of accuracy. Their formulae (equations 1–8) and coefficients (their tables 1 and 2) were implemented in R software (R Core Team, version 4.5.2) and used to create L-, M-, and S-cone opsin sensitivity functions for wavelengths between 360 and 830 nm in 1-nm increments. Note that the L-opsin spectrum is a mixture of the two L-opsin phenotypes found in the general population, L(ala180) and L(ser180), in the ratio 44%:56%. To generate a melanopsin (MEL) sensitivity spectrum, the Stockman and Rider M-opsin sensitivity spectrum was shifted (in log wavelength space) such that the peak wavelength (λ_max_) resided at 479 nm.^24^ To create age-specific crystalline lens pigment density spectra, equation 1 (with coefficients from table 5 in Stockman et al., 1999^25^) was used from van de Kraats et al. (2007),^26^ and the resulting lens density spectrum replaced lens(λ) in Stockman and Rider 2023’s equation 7. The eye of a 10-year-old child was modeled, which represents a biologically critical period for the development of refractive errors, such as myopia (nearsightedness), that typically occur between the ages of 6 and 12 years. However, the lens density changes with age and mostly prior to young adulthood. Crystalline lens density estimates are imperfect and choosing one for a child younger than 10 years old would increase its uncertainty. The optical density scaling values were −0.38, 0.38, 0.3, and 0.3 for L, M, S, and MEL, respectively.^23^ Similarly, crystalline lens scaling was 1.0 for all channels, whereas the scaling values for the macula were 0.271, 0.271, 0.271, and 0.0.

**Table 1.** The Solstice and Equinox Dates, Along With the Time at Which Daylight Breaks (Marked by the End of Morning Civil Twilight, at the Latitude of Kongsberg, 59.67°N), the Hours of Daylight and Darkness Averaged Over 4 Years (September 2022 to July 2025)

Solstice/Equinox	Dates	Clock Time for When Daylight Breaks, h:min	Hours of Daylight, h:min	Hours of Darkness, h:min
Winter	December 21	09:20	05:59	18:01
Spring	March 18	06:29	12:02	11:58
Summer	June 21	04:01^*^	18:44	05:16
Autumn	September 25	07:12^*^	12:00	12:00

The hours of daylight are calculated from the end of morning civil twilight to the start of evening civil twilight, when the sun is approximately 6 degrees below the horizon at each instance.

Times marked with ^*^ are in Daylight Saving Time.^33^

**Table 2.** Mean Alpha-Opic EDI Based on Estimated Marginal Means (EMMs), 95% Confidence Intervals (CIs), and Tukey-Adjusted *P* Values for Pairwise Comparisons (Summer as Reference)

	S-Opic Illuminance, lux		MELanopic Illuminance, lux		L-Opic Illuminance, lux	
	Mean	95% CI	Mean	95% CI	Mean	95% CI
Summer
Morning	20,417	11,220–37,154	21,380	11,749–38,905	21,878	12,023–39,811
Midday	29,512	16,218–53,703	30,200	16,596–54,954	31,623	17,378–57,544
Afternoon	16,218	8,913–29,512	16,596	9,120–30,200	16,982	9,333–30,903
Autumn
Morning	4,786^*^	2,630–8,710	4,898^**^	2,692–8,913	4,898^**^	2,692–8,913
Midday	12,303	6,761–22,387	12,589	6,918–22,909	13,183	7,413–23,988
Afternoon	3,020^**^	1,660–5,495	3,020^**^	1,660–5,370	2,884^**^	1,585–5,248
Winter
Morning	1,585	871–2,818	1,445	794–2,630	1,148	631–2,089
Midday	3,388^***^	1,862–6,026	3,162^***^	1,738–5,754	2,754^***^	1,514–5,012
Spring
Morning	11,482	6,310–20,417	11,220	6,166–20,417	10,715	5,888–19,055
Midday	21,380	11,749–38,905	21,380	11,749–38,905	20,893	11,482–38,019
Afternoon	3,548^**^	1,950–6,457	3,388^**^	1,862–6,166	3,020^**^	1,660–5,495

* *P* value < 0.05.

** *P* value < 0.01.

*** *P* value < 0.001.

### Conversion From Irradiance to Photopic Illuminance and Alpha-Opic Equivalent Daylight Illuminance

Data will be presented as total irradiance [µW/cm^2^], photopic illuminance [lux], and alpha-opic EDI [lux]. Absolute irradiance data were transformed to photopic or alpha-opic EDI using their respective spectral sensitivity functions. Photopic illuminance requires the photopic luminous efficiency function, V*(λ), which describes the sensitivity of the human eye to different wavelengths under photopic conditions.^27^ This curve is the weighted combination (1.55:1, see equation 3 in Stockman et al., 2000)^28^ of the responses of the age-specific L- and M-cone spectral sensitivities and peaks around 555 nm. Alpha-opic EDI requires the spectral sensitivity functions for the opsins contained in each of the S-, M-, and L-cone photoreceptors as well as the melanopsin-containing photoreceptors (ipRGCs).

**Figure 1.**
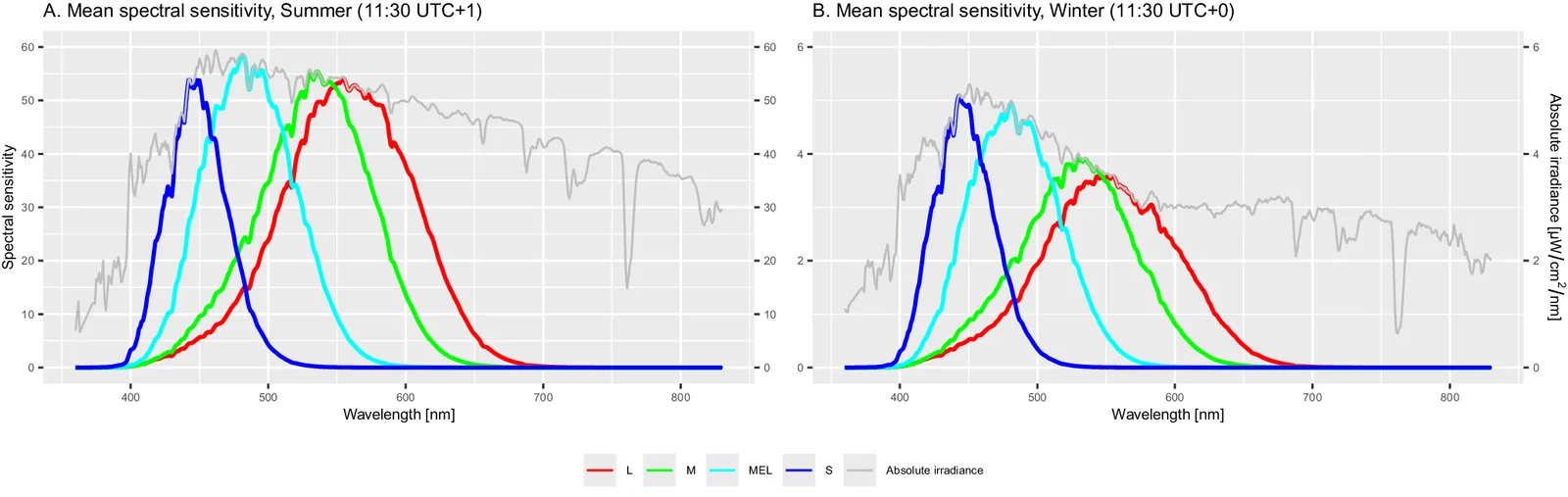
Mean absolute irradiance and the computed photoreceptor sensitivities for midday at the summer (**A**) and winter solstices (**B**), for a 10-year-old child's S-, MELan-, M-, and L-opic photoreceptors. Based on measures within the 360 to 830 nm wavelength range, averaged over a 9-day window centered on the solstice dates, and averaged over 4 years. The short-wavelength sensitive photoreceptors (S and MEL) are noticeably more activated than M and L in winter compared to summer.

The absolute irradiance at each wavelength is multiplied by each photoreceptor's spectral sensitivity at the corresponding wavelength to yield the opsin-specific power sensitivity distribution (PSD), and the area under this distribution was summed to obtain either the total photopic illuminance (using V*(λ) as the spectral sensitivity function) or alpha-opic EDI (using the opsin-specific sensitivity functions). These quantities represent the illuminance as perceived by the human eye and by each of the photoreceptors (Figs. 1A, 1B). The alpha-opic EDI conversion functions were based on, and verified against, the Python-based LuxPy color science toolbox.^29^

### Daylight Data: Time of Year and Day

Data presented here are centered around the solstice and equinox dates marking the change of seasons at this latitude (59.67°N; Table 1). Winter solstice marks the shortest day and longest night of the year; spring equinox where day and night are most equal in length, signaling the start of spring; summer solstice marks the longest day and shortest night of the year; autumn equinox where day and night are most equal in length, signaling the start of autumn. The times at which daylight breaks, as well as the number of hours of daylight and darkness for the solstice and equinox dates are also included in Table 1.

All times throughout this paper are presented in local time. The transition to and from daylight savings time occurs on the last Sunday of March (to summertime, UTC+1 to UTC+2) and the last Sunday of October (to wintertime, UTC+2 to UTC+1), both of which occur more than 2 weeks after their respective (spring and winter) equinoxes.

To reduce the influence of varying weather patterns on the specific days of interest, our analysis included data over a 9-day window centered on each solstice and equinox date, and, because the measured irradiance inherently reflects contemporaneous weather conditions and our focus was on seasonal rather than day‑to‑day variability, we did not include detailed daily meteorological data for each measurement day. Only specific periods of the day were analyzed, which corresponded to expected outdoor time for a 10-year-old school child. These periods included: arrival in the morning and the first outdoor recess (08:00–08:10 and 10:00–10:15); midday (11:30–12:00 and 13:00–13:10); and departure in the afternoon along with outdoor activity after school (16:00–16:10). Typically, after school, children are reported to spend approximately 1 hour on sports or outdoor activities (18:00–19:00). The combination of afternoon outdoor recess and after-school outdoor activities was considered as late afternoon outdoor time for subsequent analysis.^30^ The cumulative exposure and standard deviation for each of these three time periods (25 minutes, 30 minutes, and 70 minutes, averaged over the 9-day period) were computed. Note that winter measurements before 09:20 and after 15:19 are excluded from the analysis because these are during periods of darkness. Similarly, only the measurements before 18:31 (before sunset) were included in the 18:00 to 19:00 spring recess.

### Participants and Ocular Measurements

To infer whether the eye may use light intensity and/or spectral composition to assist in the regulation of eye growth, ocular measurements (that were obtained at 4 time-points during 2019–2020 and at the same geographic location as the daylight measurements) were reanalyzed. Importantly, this constitutes a comparison of typical seasonal patterns, not an analysis of the exact environmental conditions experienced by the cohort. The following measurements were obtained: cycloplegic autorefraction (Huvitz HRK-8000A, Huvitz Co. Ltd., Gyeonggi-do, Korea), non-cycloplegic autorefraction (Nvision-K 5001 open-view autorefractor, Shin-Nippon, Tokyo, Japan) at distance (600 cm), and ocular biometry to and axial length (IOLMaster 700; Carl Zeiss Meditec AG, Jena, Germany). Ninety-two children (44 girls; aged 7–11 years) were enrolled and followed over a 12-month period. The first set of measurements were collected in late November 2019, followed by repeated measurements 7 weeks later in January 2020 (spaced roughly equally before and after winter solstice), and a third repeat 20 weeks later in early June (2 weeks prior to summer solstice). The final measurements were obtained the following November. Only data for the 65 children who had persistent emmetropia or mild hyperopia (*n* = 30, 7–8-year-olds and *n* = 35, 10–11-year-olds) were included in the analysis presented here. The children spent a minimum of 1 to 2 hours outdoors on school days, as outdoor time during recess is compulsory at this school irrespective of the time of year. The details of the data gathering protocol are given in Nilsen et al. (2023).^18^

This part of the study was approved by the Regional Committee for Medical and Health Research Ethics (Southern Norway Regional Health Authority) and both parents/caregivers provided written consent for their child to participate. The study was carried out in accordance with the tenets of the Declaration of Helsinki. All children included in the study were healthy with no history of ocular disease as reported by their parents.

### Comparisons Between Roof-Mounted and Eye-Height Sensors

Five Actlumus (Condor Instruments, São Paulo, Brazil) light loggers were attached to a tripod set to a height of 135 cm (average eye-height of a 10-year-old child in the Nilsen et al., 2023 study)^18^ and positioned in their school playground. One light logger was oriented skyward to provide a direct comparison with the Ocean Optics roof-mounted spectrometer. The other four light loggers were oriented toward the north, east, south, and west horizons, respectively, to represent the field of view of a typical child facing in any of those directions. The light loggers recorded 7 minutes of absolute irradiance data between 15:00 and 15:07 on May 25, 2026, during an overcast sky, which is beneficial when comparing light sensors.^31^ The photopic illuminance measurements were compared with those from the Ocean Optics spectrometer for the same period.

### Statistical Analysis

The analysis was conducted using the lmer function from the lme4 package in R software (version 4.5.2) to fit a linear mixed-effects model to assess within channel variation across seasons and time of day. The factor season represents spring equinox, summer solstice, autumn equinox, winter solstice. Channel represents photopic illuminance and equivalent daylight illuminance for the cone opsins and melanopsin, in lux, respectively, as well as alpha-opic EDI ratios (e.g., S:L and S:MEL). Recess represents different time periods during the day (morning, midday, and afternoon).

Likelihood ratio tests were used to compare nested models and assess the significance of interaction terms and main effects. Models with and without the three-way interaction term (season:recess:channel) were compared using the anova() function in R software. All terms were found to be significant and were retained. The contribution of individual predictors was further assessed using the drop1() function. Additionally, type II Wald chi-square tests were performed using the Anova() function from the car package, to assess the significance of fixed effects. Fixed effects included channel, the interaction between channel and recess, and among channel*,* recess, and season. Random effects accounted for measurement days where year_season_day uniquely identified each of the 144 days analyzed (9 days per season over 4 years), and data contained the mean illuminance (mean) over each recess for each channel, day-of-9-day-window, season, and year (3 × 13 × 9 × 4 × 4 values, respectively). The final model included all predictors along with three-way interactions, and estimated coefficients only for the individual levels of the fixed effects. It was specified as:

$$\begin{array}{ccc} & & \mathrm{lmer}(\mathrm{mean}∼\mathrm{channel}/\mathrm{recess}/\mathrm{season}\\ & & \;+\;(1|\mathrm{year}/\mathrm{season}/\mathrm{day})\;+\;0,\mathrm{data}) \end{array}$$

Post hoc pairwise comparisons of estimated marginal means were conducted using the emmeans package, stratified by channel and recess, to investigate specific contrasts within channel variation between summer and the three other seasons for each recess. To control for type I errors, *P* value adjustments for multiple comparisons were applied using the Tukey method.

Under the null hypothesis, that there was no difference between the conditions, residual diagnostics were performed to evaluate the assumptions of the model. Residuals versus fitted values were plotted to assess homoscedasticity, and a Q-Q plot with a reference line was used to evaluate the normality of residuals. For the alpha-opic EDI ratios, both mean and cumulative, the Q-Q plots indicated heavy-tailed deviations from normality, suggesting that the residuals did not meet the assumption of normality required for accurate inference. To assess the robustness of the results, a permutation test^32^ was conducted, which does not rely on distributional assumptions. The permutation test involved shuffling the response variable and recomputing the test statistics across 10,000 iterations to generate a null distribution, allowing us to assess the robustness of the observed *P* values.

## Results

### Seasonal Variation in Intensity and Illuminance During the Day

**Figure 2.**
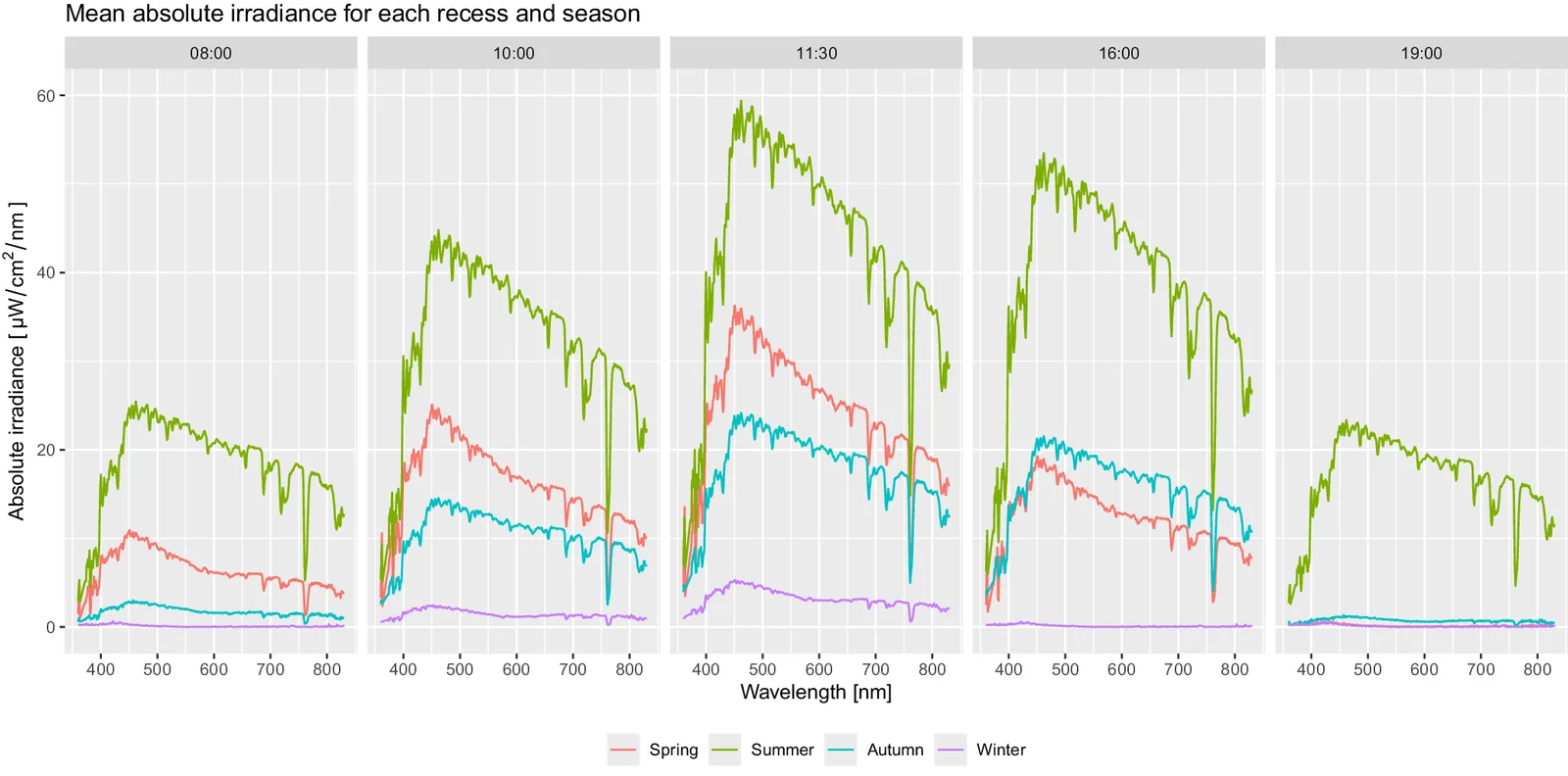
Mean absolute irradiance as a function of wavelength (360–830 nm) for each season at 5 different local times during the day averaged over a 9-day window centered on each equinox/solstice and averaged over 4 years. Absolute irradiance increases with increasing wavelength from 350 nm with a peak around 450 nm followed by a decline toward longer wavelengths. Overall irradiances (area under the curve) increase from morning to midday, followed by a subsequent decline in the afternoon. This pattern is also present in winter, with daybreak at 09:19 and darkness falling at 15:17, resulting in lower irradiance levels in the morning, midday, and afternoon. Evidence of the blue-hour, where short-wavelengths are proportionally more intense than other wavelengths, can be seen in winter measurements and also at 08:00 during autumn and spring.

**Figure 3.**
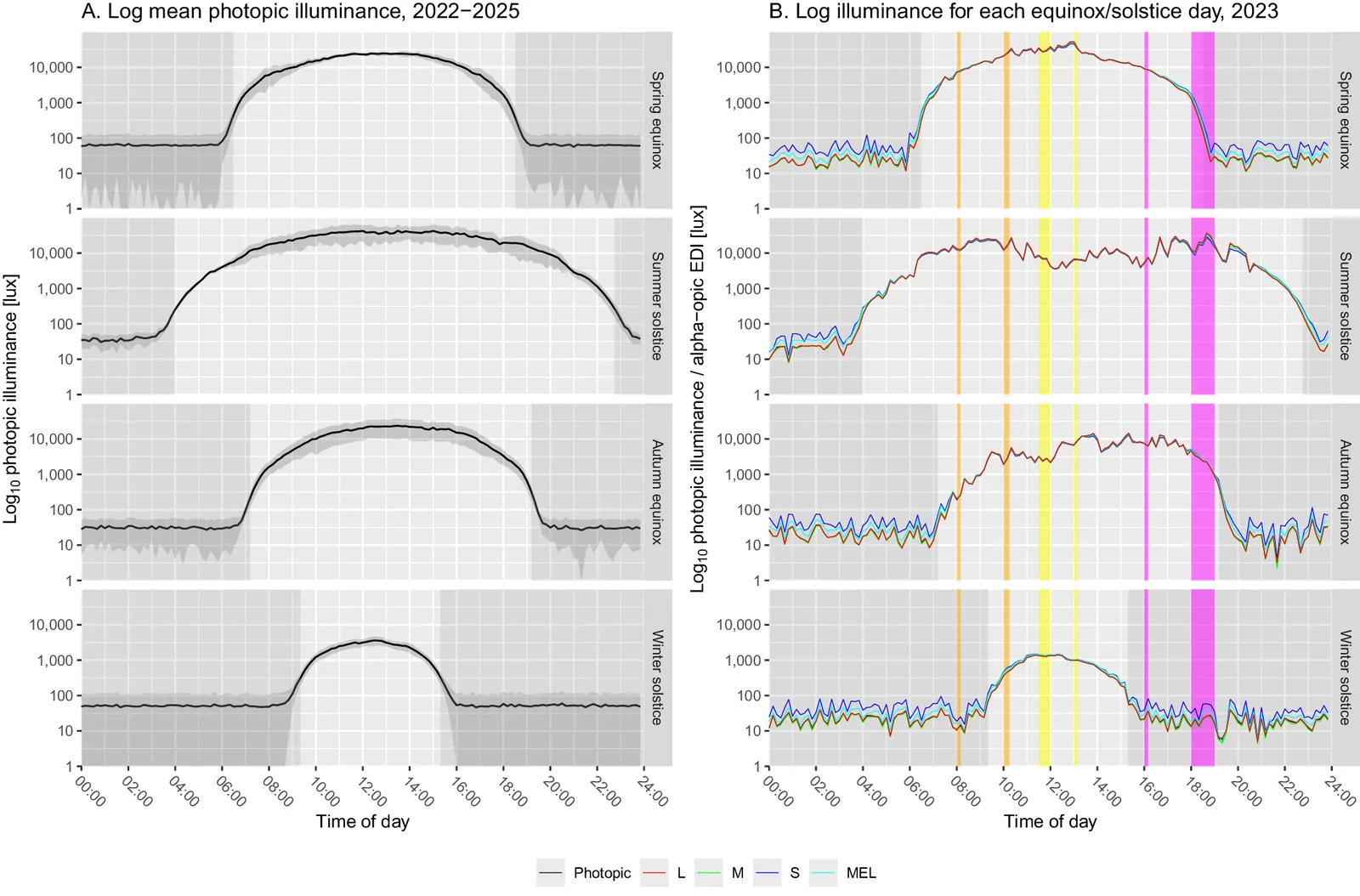
Log photopic illuminance and alpha-opic EDI (lux) for every 10th minute of the day. Each row is based on the mean of a 9-day window of measurements centered on the respective equinox/solstice. (**A**) Log mean photopic illuminance and 95% confidence intervals over the years 2022 to 2025. (**B**) Variations in illuminance levels for a representative year (2023), showing photopic (*black line*) and alpha-opic EDI for the S-, M-, and L-opsins (*blue*, *green*, and *red lines*), as well as melanopsin (MEL, *cyan line*). Recess and outdoor times are marked by *yellow* (morning), *blue* (midday), and *purple* (afternoon) bands. The period from evening to morning civil twilight (defined as the sun being approximately 6 degrees below the horizon at each instance) is marked by grey bands. Based on measures within the 360 to 830 nm wavelength range, calculated for a 10-year-old child.

Figure 2 depicts the mean absolute irradiance at 5 time points throughout the day across 4 seasons and 4 years. Figure 3 shows the calculated log mean photopic and alpha-opic EDI weighted for the opsins S, MEL, and L in the eye of a 10-year-old child as a function of the time of day.

**Figure 4.**
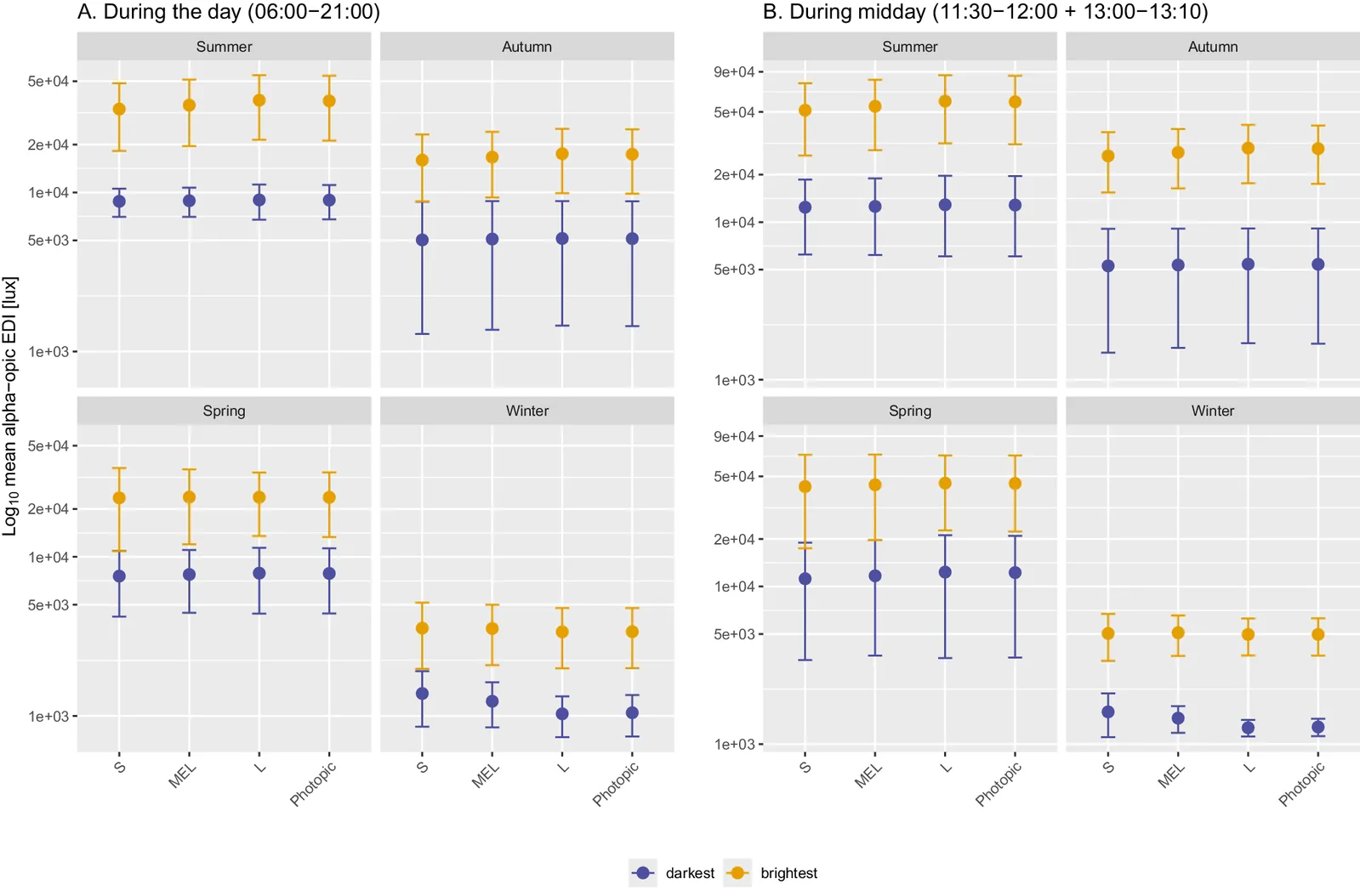
Darkest and brightest days across seasons. Mean photopic illuminance and alpha-opic EDI during daytime (**A**), 06:00 to 21:00 or sunrise to sunset whichever is shortest and midday (**B**), 11:30 to 12:00 and 13:00 to 13:10 across 4 years by season for S-, MEL-, and L-opsin on the darkest and brightest days. Based on measures within the 360 to 830 nm wavelength range, calculated for a 10-year-old child.

Figure 4 shows the variation in available average photopic illuminance and alpha-opic EDI on the brightest and darkest days over typical waking hours for Norwegian school children (between 06:00 and 21:00 or sunrise to sunset whichever is shortest; see Fig. 4A) and during midday recesses (see Fig. 4B), across the four seasons and years. Mean daytime illuminance on bright spring and autumn days was typically 15,000 lux or higher, with spring days being brighter. Dark spring and autumn days were approximately 5000 lux. Mean illuminance on bright winter days was approximately 3700 lux, occasionally reaching 5000 lux midday, whereas on dark days it was 1000 lux, even at midday. Mean illuminance on bright summer days is approximately 35,000 lux, occasionally reaching 85,000 lux midday.

### It Is Dark During Winter School Mornings and Afternoons

Children do not spend the entire day outdoors; therefore, it is useful to determine the amount of daylight they could be exposed to during the periods they are typically expected to be outside. To address this, we estimated both mean and cumulative alpha-opic illuminances available during the times of day when population-level evidence suggests that school-aged children are likely to be outdoors. The mean represents a near-instantaneous (e.g., across a recess) measurement, whereas the cumulative value is more informative when considering the eye in a dose-response relationship with daylight.

Tables 2 and 3 presents the mean EDI values for each of the S-, MEL-, and L-opic illuminances.

**Table 3.** Cumulative Alpha-Opic EDI Based on Estimated Marginal Means (EMMs), 95% Confidence Intervals (CIs), and Tukey-Adjusted *P* Values for Pairwise Comparisons (Summer as Reference)

	S-Opic EDI, lux		MELanopic EDI, lux		L-Opic EDI, lux	
	Mean	95% CI	Mean	95% CI	Mean	95% CI
Summer
Morning	512,861	281,838–933,254	524,807	288,403–977,237	549,541	301,995–1,000,000
Midday	1,174,898	645,654–2,137,962	1,230,269	676,083–2,238,721	1,258,925	691,831–2,290,868
Afternoon	1,148,154	630,957–2,089,296	1,174,898	645,654–2,137,962	1,202,264	660,693–2,187,762
Autumn
Morning	120,226^**^	66,069–218,776	123,027^**^	67,608–218,776	123,027^**^	67,608–223,872
Midday	489,779	269,153–891,251	512,861	281,838–912,011	537,032	295,121–977,237
Afternoon	208,930^**^	114,815–380,189	208,930^**^	114,815–380,189	199,526^**^	109,648–363,078
Winter
Morning	22,909^***^	12,882–41,687	21,380^***^	11,749–38,905	16,982^***^	9,333–30,903
Midday	134,896^***^	74,131–245,471	125,893^***^	69,183–229,087	109,648^***^	60,256–199,526
Spring
Morning	281,838	154,882–512,861	281,838	154,882–501,187	263,027	144,544–478,630
Midday	851,138	467,735–1,548,817	851,138	467,735–1,548,817	831,764	457,088–1,513,561
Afternoon	144,544^***^	79,433–263,027	138,038^***^	75,858–251,189	123,027^***^	67,608–223,872

* *P* value < 0.05.

** *P* value < 0.01.

*** *P* value < 0.001.

**Figure 5.**
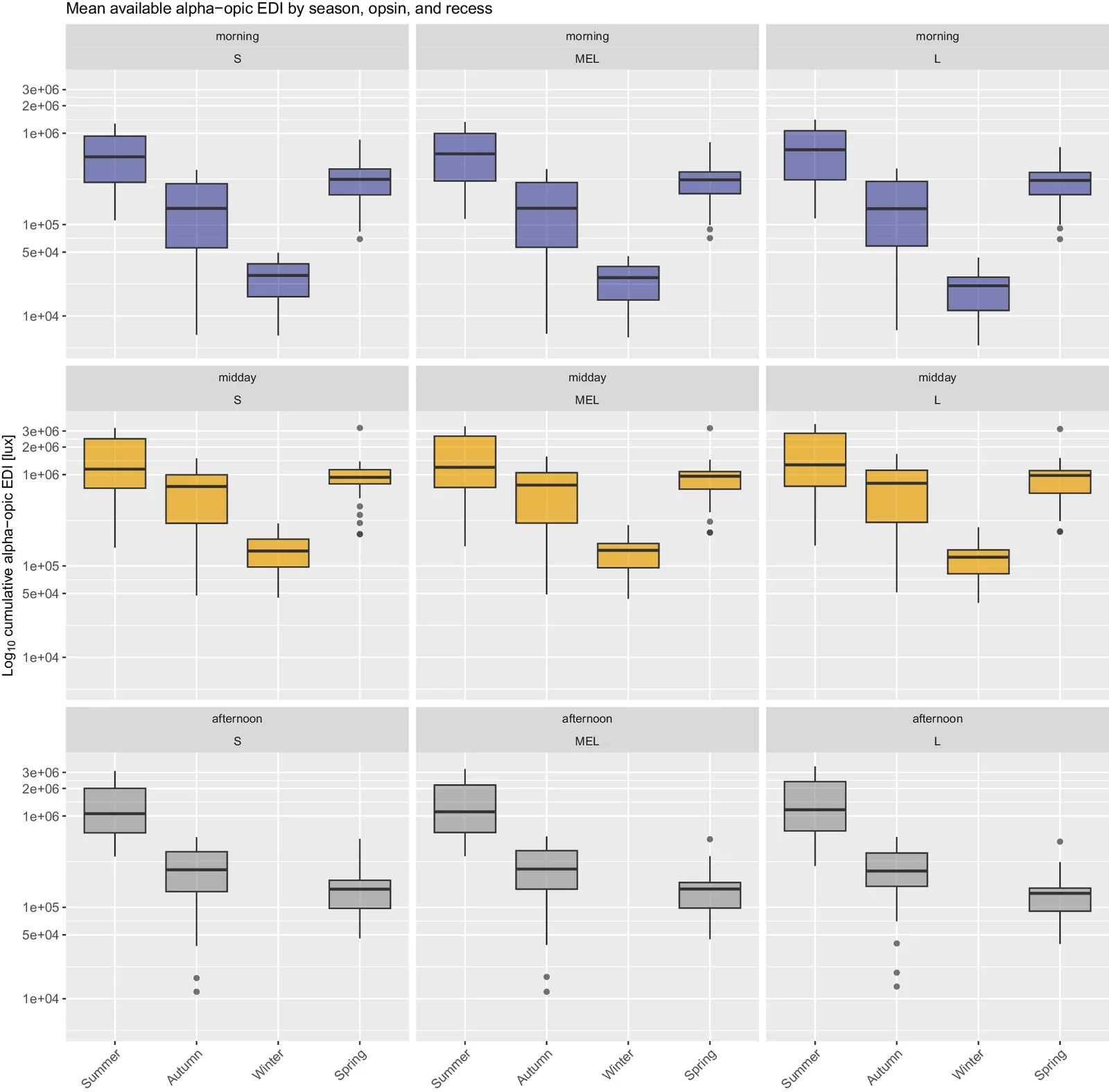
Mean available alpha-opic EDI during morning and midday outdoor recess, as well as late afternoon outdoor activities for S-, MEL-, and L-opsin. Measurements were averaged over 9-day windows centered on each equinox/solstice date, based on 360 to 830 nm wavelengths and calculated for a 10-year-old child. Summer is shown on the *left* as this is the reference in the statistical analysis.

In Figure 5, mean alpha-opic EDI are shown for outdoor time during the morning, midday, and late afternoon for S-, MEL-, and L-opsin across four seasons. The respective cumulative alpha-opic EDI are shown in Supplementary Figure S1.

The three-way interactions revealed significant variations in mean cone-opic illuminance within cone type across seasons and time of day. When comparing with summer, mean S-, and MEL- and L-opic illuminance were significantly lower in autumn (adjusted [adj.] *P* < 0.01) and winter during morning (adj. *P* < 0.001), winter during midday (adj. *P* < 0.001), as well as in autumn (adj. *P* < 0.01) and spring during afternoon (adj. *P* < 0.001). Afternoon after-school outdoor activities were after sunset during winter. The same variations were observed for cumulative cone-opic illuminances.

### School Days Are Dim and Blue Enriched in Autumn, Winter, and Spring, Whereas Balanced White in Summer

To answer the question if there is a difference in relative levels of short- (blue) versus long- (red) wavelength daylight in winter compared with summer, we calculated the ratios of S:L, MEL:L, and L:M, as well as (S+MEL):(L+M) alpha-opic EDI.

Table 4 presents the mean EDI ratios for each of the S-, MEL-, and L-opic illuminances. The respective cumulative alpha-opic EDI ratios are shown in Supplementary Table S1.

**Table 4.** Mean Alpha-Opic EDI Ratios Based on Estimated Marginal Means (EMMs), 95% Confidence Intervals (CIs), and Tukey-Adjusted *P* Values for Pairwise Comparisons (Summer as Reference)

	S:L Ratio		MEL:L Ratio		S+MEL:L+M Ratio		L:M Ratio	
	Mean	95% CI	Mean	95% CI	Mean	95% CI	Mean	95% CI
Summer
Morning	0.94	0.88–1.00	0.96	0.90–1.03	0.96	0.90–1.02	1.01	0.94–1.08
Midday	0.93	0.87–0.99	0.96	0.89–1.02	0.95	0.89–1.01	1.01	0.94–1.08
Afternoon	0.98	0.91–1.04	0.99	0.93–1.06	0.99	0.92–1.05	1.01	0.94–1.07
Autumn
Morning	1.05	0.98–1.12	1.04	0.97–1.11	1.04	0.97–1.11	0.99	0.93–1.09
Midday	0.92	0.86–0.98	0.95	0.89–1.02	0.95	0.89–1.01	1.02	0.95–1.03
Afternoon	1.17^*^	1.09–1.25	1.12^*^	1.05–1.21	1.12^*^	1.05–1.20	0.96	0.90–1.01
Winter
Morning	1.36^***^	1.28–1.46	1.25^***^	1.17–1.33	1.27^***^	1.19–1.35	0.94	0.88–1.01
Midday	1.24^***^	1.16–1.32	1.16^**^	1.09–1.24	1.18^**^	1.10–1.26	0.96	0.90–1.03
Spring
Morning	1.12	1.05–1.19	1.08	1.01–1.15	1.09	1.02–1.16	0.98	0.92–1.05
Midday	1.02	0.96–1.09	1.02	0.96–1.09	1.02	0.96–1.09	1.0	0.93–1.06
Afternoon	1.56^***^	1.46–1.67	1.37^***^	1.29–1.47	1.40^***^	1.31–1.50	0.92^*^	0.86–0.98

* *P* value < 0.05.

** *P* value < 0.01.

*** *P* value < 0.001.

**Figure 6.**
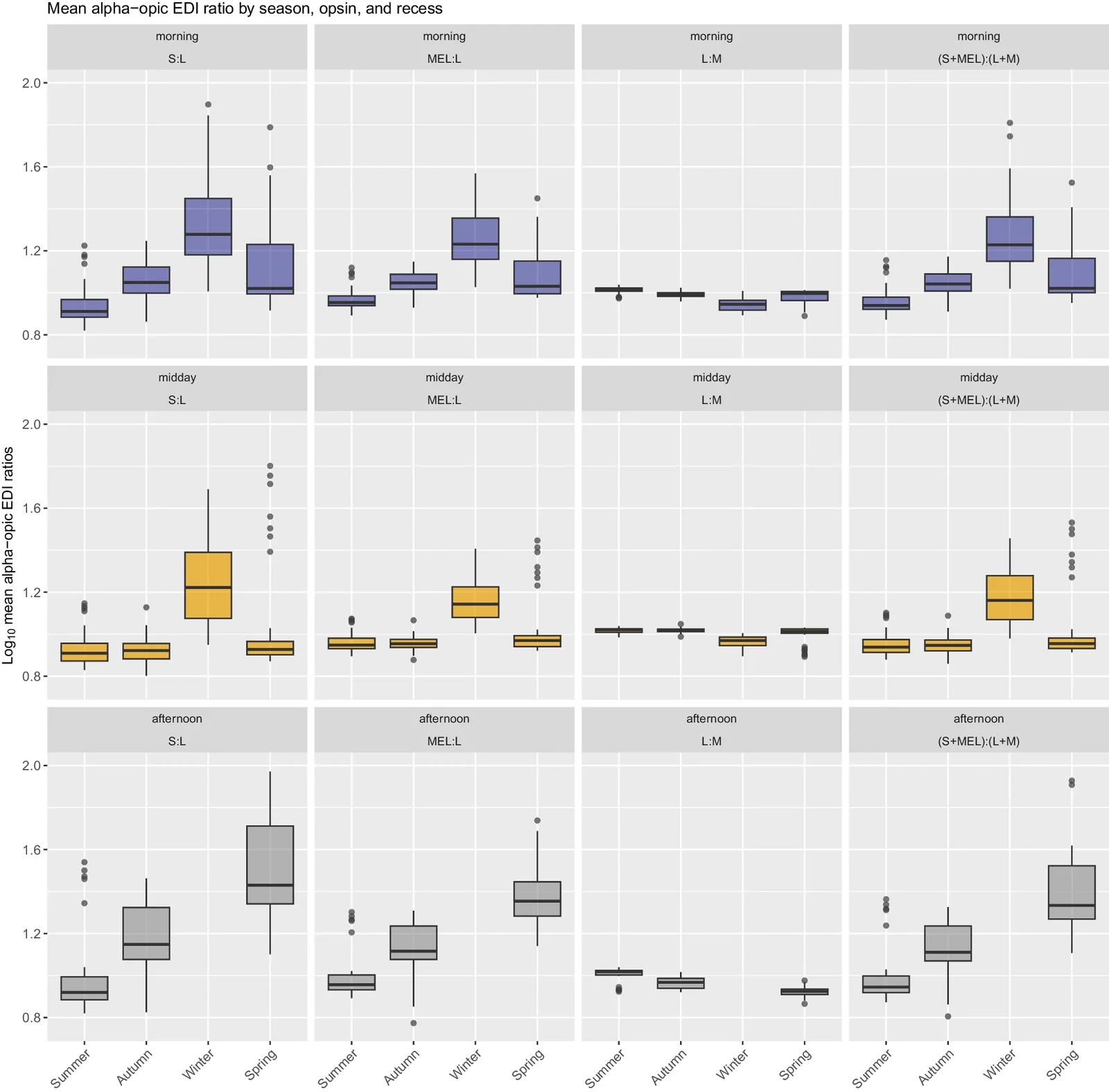
Average available alpha-opic EDI ratios during morning and midday outdoor recess, as well as late afternoon outdoor activities for S:L, MEL:L, L:M, and (S+MEL):(L+M) opsin ratios. Measurements were averaged over 9-day windows centered on each equinox/solstice date, based on 360 to 830 nm wavelengths and calculated for a 10-year-old child. Summer is shown on the *left* as this is the reference in the statistical analysis.

Figure 6 shows the mean alpha-opic EDI ratios. The S:L, MEL:L, and (S+MEL):(L+M) ratios are higher in winter compared with summer, whereas L:M ratios remain stable across seasons. The respective cumulative alpha-opic EDI ratios are shown in Supplementary Figure S2.

A linear mixed-effects model was fitted to each of the mean and cumulative cone-opic illuminance ratios. The three-way interactions revealed significant variations in mean and cumulative ratios of cone-opic illuminance across seasons and time of day.

#### Mean Ratios

When comparing with summer, the mean ratios of S:L, MEL:L, and (S+MEL):(L+M) were significantly larger during the afternoon in autumn (all adj. *P* < 0.05) and spring (all adj. *P* < 0.001), as well as during morning (all adj. *P* < 0.001) and midday (all adj. *P* < 0.01) in winter. When assessing statistical significance using permutation test, all *P* values remained significant, except for the comparison during the afternoon in autumn.

#### Cumulative Ratios

When comparing with morning, the cumulative ratios of S:L, MEL:L, and (S+MEL):(L+M) were significantly larger during the morning (all adj. *P* < 0.001) and midday (all adj. *P* < 0.01) in winter as well as during the afternoon in spring (all adj. *P* < 0.05). When assessing statistical significance using permutation test, all *P* values remained significant. The respective figure and table for cumulative alpha-opic EDI ratios are shown in Supplementary Figure S2 and Supplementary Table S1.

### Regulated Eye Growth Varies Across Seasons

**Figure 7.**
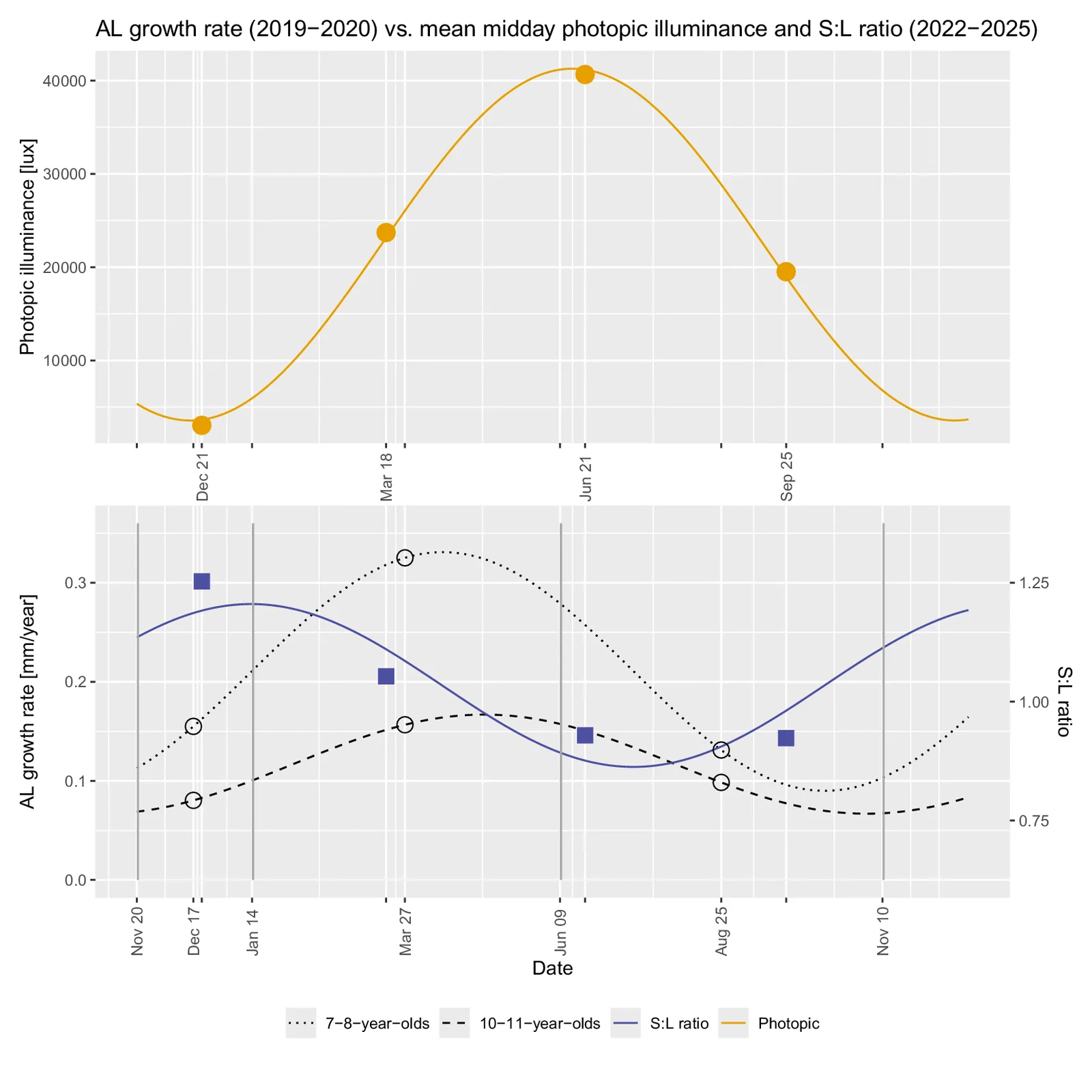
Seasonal variation in photopic illuminance (*top panel*, *solid orange line*), S:L EDI ratio (*lower panel*, *solid blue line*), and ocular axial growth rate in 7 to 8- and 10 to 11-year-old children (*dotted* and *dashed lines*, respectively). Ocular axial length was measured approximately November 20, January 14, June 9, and November 10 (*vertical dark-gray lines*). The sinusoidal fit of seasonal variation in ocular axial growth rate assumes that peak growth rate (mm/month) lies midway between the January and June measurement points.

The children who remained emmetropes or low hyperopes throughout the year were assumed to experience normal (physiological) eye growth. Figure 7 shows the sinusoidal fit of seasonal variation in eye growth rate, under the assumption that the peak growth rate (mm/year) occurs midway between the winter and summer measurements; this function (dashed and dotted lines) corresponds well with the observed data (open circles). It indicates that during the period of the year with the shortest days and lowest light levels (November–January), growth rates are increasing and continue to rise into the spring. From spring through summer (June to approximately October), growth rates decline before increasing again in November.

### Comparisons Between Roof-Mounted and Eye-Height Sensor

**Figure 8.**
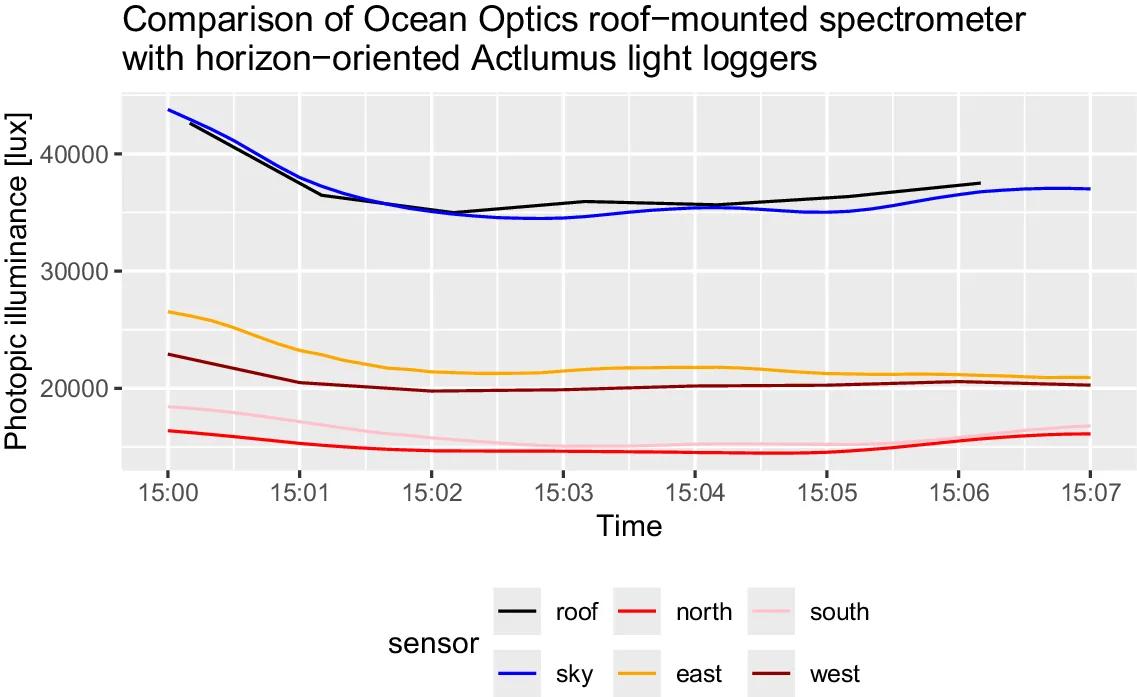
Comparison of Ocean Optics roof-mounted spectrometer (“roof”) with 5 Actlumus light loggers mounted at the eye height of a 10-year-old child. Four of the Actlumus devices were oriented toward the north, east, south, and west horizons, whereas the fifth was oriented skyward (“sky”) to provide a direct comparison with the Ocean Optics sensor. There was good agreement between illuminance measurements from the “roof” and “sky” sensors, whereas the four horizon-facing sensors measure proportionally less illuminance (north/sky, east/sky, south/sky, and west/sky measure 42%, 55%, 42%, and 53% of downwelling illuminance, respectively).

Figure 8 shows the photopic illuminance recorded by the five Actlumus light loggers and the Ocean Optics roof-mounted spectrometer during an overcast sky.^31^ There is good agreement between the roof-mounted sensor (“roof”) and the sky-ward pointing Actlumus logger (“sky”), suggesting the Actlumus records a representative value of photopic illuminance despite the differences in sensor technologies. The north-, east-, south-, and west-facing Actlumus loggers recorded proportionately less absolute photopic illuminance, as would be expected from the loggers' orientations, but also due to the absorption of light by nearby school buildings, trees, and the surrounding urban environment. The mean ratios of the north/south/east/west loggers relative to the sky logger were north/sky = 0.42; south/sky = 0.42; east/sky = 0.55; and west/sky = 0.53.

## Discussion

We documented seasonal and diurnal variation in available daylight intensity and spectrum at a 60°N latitude location. Downwelling absolute irradiance measurements were converted to ratios of alpha-opic EDI for the S-, MEL-, M-, and L-opsin sensitivities, along with ocular media transmission for a 10-year-old child. A key observation was the pronounced reduction in available illuminance during mornings and midday through winter and spring compared with summer, as well as during autumn and spring afternoons compared with summer, and that during these times, the short-wavelength intensity was higher relative to long-wavelength intensities. This underlines that the S cones and ipRGCs have more light available for stimulation than the M and L cones at certain times of the day and the year. Concurrently, the bulk of regulated eye growth in 7 to 11-year-old children growing up at this latitude begins in early winter and is fastest in late winter to spring. This period appears to be delayed relative to the period with the shortest days when natural daylight is limited in time and low in intensity and continues through spring when daylight increases in length and intensity but remaining more intense in short wavelengths. This opens the possibility that the annual solar cycle could influence the regulation of human eye growth at this latitude.

The dimming of intensity and spectral shifts reflect changes in solar angle and atmospheric scattering, which increase the relative contribution of shorter wavelengths. Comparable measurements have been reported at the same latitude.^1^ At 60°N, there is a 13-hour difference in daylight duration between winter and summer. The solar angle in winter is −8 degrees at the start of the school day and at 7 degrees midday, whereas in summer, it is 25 degrees at the start of the school day and 54 degrees midday.^33^ The children at the study location typically walk to school throughout the year, which will be at times of dark or dusk during winter. The available average illuminance levels at this time of year during expected waking hours for a 10-year-old school child, are in the range <1000 to 6000 lux (see Table 2), although a child will typically receive half of this (see Fig. 8). When coupled with the fact that after-school outdoor activities occur after sunset in winter, it implies limited availability of daylight during critical times of the day. That the outdoor illuminance levels during winter can be below 1000 lux questions the commonly used threshold of 1000 lux as a proxy for being outdoors.^4^^,^^34^ Similar indications are reported in other studies, both for humans and animals. He and colleagues^35^ used light-sensing smartwatches to assess personal light exposure in 5 to 9-year-old children. They showed that approximately 2 hours of exposure to mean 5000 lux per day (equivalent to a daily dose of 600,000 lux) only reduced the myopia incidence risk by 15%. Children growing up at 60°N will not reach such an exposure dose in winter (see Table 3). Ashby and colleagues have demonstrated that, in chicken models, experimentally induced myopia can be inhibited by exposure to 15,000 lux or higher,^11^^,^^36^^,^^37^ and as long as the spectrum is relative broad, the spectral composition appears not to play a role.^11^ However, for lower intensities, blue-enrichment may be required for slowing eye growth and speed up recovery from experimentally induced myopia in chicken models.^38^^,^^39^

One-third of the growth necessary for the human eye to achieve its adult size occurs postnatally and continues through to the end of adolescence.^40^^,^^41^ Both regulated and dysregulated myopic eye growth (grows too much) is reported to vary with season.^16^^–^^18^ Prevailing theories of myopia development suggest that near-work alone is unlikely to be a major causal factor in dysregulated eye growth; instead, near-work appears to become problematic primarily when combined with insufficient time spent outdoors.^5^ In the present study, during the period in which annual eye growth was assessed, these children had 17 more school days during the winter compared with the summer observation period,^18^ making it plausible that children experienced greater academic load and near work in winter. This increased academic load and near work would have occurred in addition to outdoor time under low light intensity levels assumed to contribute to accelerated eye growth. By 2019 to 2020, the children included in the study (7–8- and 10–11-year-olds) had accumulated approximately 5 and 10 years in structured educational settings. In Norway, more than 92% of children aged 1 to 5 years and over 97% of those aged 3 to 5 years attend preschool.^42^ Children start school in the calendar year in which they turn 6 years old; thus, children aged 7 to 8 and 10 to 11 years will have completed 1 to 2 and 4 to 5 years of primary education. Considering that Norwegian children spend more time on digital screens outside of school than their peers across Europe,^43^ and if screen time serves as a proxy for reduced outdoor activity outside of school, one might expect that most children growing up at the 60°N latitude (where the irradiance measurements were obtained) would exhibit accelerated eye growth (too much growth) in autumn and winter, resulting in a correspondingly high prevalence of myopia. However, the prevalence of myopia in this region of Norway and in Nordic countries remains low and stable,^19^^–^^22^ which is in contrast to what is observed in East-Asian countries that are closer to the equator.^6^ The majority of children growing up in Norway, at 60°N latitude, experience most of their annual eye growth between November and June, with a peak in late winter to spring, and very few experience myopic (accelerated) eye growth.^18^ This suggests that when daylight intensity is low, the spectrum and intensity of available daylight may be integrated to provide reliable information that may contribute to the regulation of eye growth. This is the case for circadian rhythms. Integration of light intensity and spectrum through activation of both S-cones and ipRGCs is shown to enhance the stability of circadian-clock-driven rhythms under conditions of low light intensity, when timing cues are weak or ambiguous.^44^^,^^45^ DA is reported to be synthesized and released in a dose-dependent manner that is proportional to the intensity of light.^46^ A reasonable hypothesis is that integration of light intensity and spectrum, particularly under low illuminance conditions with relatively higher intensity in shorter wavelengths that activates both S-cones and ipRGCs, may result in sufficient DA release to exert minimal yet adequate inhibition, thereby supporting the regulation of eye growth in these children in autumn through winter to spring. Summer days are longer with spectrally balanced and more intense daylight, likely to provide more DA release that results in stronger inhibition and, consequently, slowing the rate of eye growth. If there is any tenacity to this, as a result, it gives rise to speculation that exposure to blue-enriched light may be beneficial during the school day to maintain regulated eye growth, if it is unlikely that the children will get exposure to spectrally balanced broadband-like daylight of 1000 lux or more. It is consistent with the hypothesis that indoor light that is as dim as a dark winter day in the Nordic region, but lacking in the short-wavelength part of the spectra, may play a role in the myopia epidemic observed in South-East Asia, which has been linked with too much indoor time and too little outdoor time with exposure to daylight.^4^

The results presented here do not contradict the theory that circadian rhythms are involved in regulating healthy eye growth.^13^ That the period of fastest eye growth is in anti-phase but delayed relative to the shortest winter days may be analogous to circadian phase shifting, where the entrainment process that averages light over weeks is slow and deliberate to ensure the eye responds in the best way to preserve fitness.^47^ It may also be analogous to the temporal integration of defocus signals in emmetropization, where the eye in different animal models of myopia has been shown to integrate visual signals over time rather than respond to instantaneous inputs, leading to a delay in the eye growth response to the visual environment.^48^^–^^51^ Further to this, downstream ocular tissues, such as the sclera and choroid, remodel themselves on relatively slow timescales.^52^ The results presented here also cannot contradict that the eye itself can assess whether it is too short or too long by utilizing longitudinal chromatic aberration,^14^ as the eye would require information about intensity and spectrum to evaluate which wavelengths of light are better focused.^15^ The results presented here also do not exclude an influence of near-work given that it was not measured directly, however, there are indications that short‑wavelength–enriched light may reduce accommodative demand and mitigate the associated biomechanical stress during indoor activities, such as sustained near work.^53^^,^^54^ Together, these findings are consistent with the hypothesis that temporal integration of retinal and downstream growth signals, combined with the intrinsic inertia of scleral and choroidal remodeling, could plausibly lead to a delayed eye‑growth response relative to seasonal changes in the light environment and/or seasonal changes in academic load combined with the light environment. Future animal and human studies will be necessary to test this hypothesis.

### Strengths and Limitations

A strength of this study is that irradiance measurements were collected and analyzed over four 9-day periods spanning 4 years, compensating for natural variability in weather, solar angle, and atmospheric factors that will affect solar radiation and the proportion of short- versus long-wavelength radiation irrespective of solar angle.^1^ The variation in local weather conditions, including cloud cover and precipitation, was not analyzed, as our focus was on seasonal rather than day‑to‑day variability.

Personal light exposure measurements were not obtained. However, this must be considered in relation to the variability among currently available wearable sensors, as well as challenges related to their form factor (e.g., pendant or spectacle-mounted), body position (e.g., wrist, chest, or head), and the potential for the sensor to be covered by clothing when outdoors in autumn, winter, and spring. Therefore, these devices, at worse, would have represented a lower-bound on the light that reached the participant's eyes.^55^ Conversely, our use of a cosine-corrected sensor for measuring downwelling irradiance assumes that the eye is looking upward, and thus represents an upper-bound to the eyes’ exposure with none of the above confounding factors. The sky-facing roof-mounted spectrometer also fails to capture the local horizon-facing environment, as different outdoor activities take place on surfaces with varying reflectance properties. Our control measures were obtained in a schoolyard at a child's eye height with light loggers placed in an open area of the schoolyard of grey tarmac with nearby areas of grass, sand, and some colored soft play-tarmac within a 100-meter radius. These measures show that the illuminance reaching the eye is approximately 50% of that measured at the roof on an overcast dry spring day. The condition of the surface, whether it is dry, wet, or covered with snow, also plays a role in determining the amount of radiation reflected toward the eye. (For a discussion about factors affecting the visual diet see Marcos, 2025^7^.) As such, if the ground is covered with fresh snow, a horizon-facing eye will, in winter, be exposed to between 50% and 90% of that measured by the sky-facing roof spectrometer. Reflectance from snow declines rapidly from approximately 90% when the snow is dry and fresh, to 40% within a day or 2, and to approximately 30% when it is contaminated or wet.^56^ Weather data from the region show similar patterns across the years 2019 to 2025, except that the winters of 2019 to 2020 had little to no snow and this was less than in the winters of 2022 to 2025.^57^ If anything, this would indicate that the chronological mismatch between irradiance measurements obtained during winters in the 2022 to 2025 period and eye growth measurements obtained in 2019 to 2020 likely led to an underestimation of the irradiance relevant to the latter. This, in turn, provides additional support for the hypothesis that when light levels are low, the eye may utilize the imbalance in short- versus long-wavelength intensity to assist in the regulation of eye growth via photoreceptor activation.

Regardless, the results should be understood as a comparison between typical seasonal light patterns at this latitude and typical seasonal eye‑growth patterns, rather than as an analysis of the precise environmental conditions experienced by the cohort. They should be regarded as contextual, population‑level indicators of ambient light, and are not suitable for drawing inferences at the level of individual children. It would be inappropriate to extrapolate the observed patterns to myopia onset or progression without further investigation.

## Conclusions

The annual cycle of day-length is the most effective synchronizer of seasonal rhythms. Considering the robustness of 4 years of real-world irradiance data following 1 year of regulated eye growth measurements, we speculate that the eye may exploit seasonal variations in light intensity and spectrum to regulate eye growth. It suggests that human eye growth may have evolved to respond to stimuli that also happen to vary seasonally at non-equatorial latitudes. That the short-wavelength shift in times of the year with low daylight illuminance encompasses the spectral sensitivities of both S-cone and melanopsin-driven ipRGC photoreceptor cells, is consistent with the possibility that the eye exploits seasonal variations in light intensity and spectrum to assist in the regulation of eye growth via photoreceptor activation. Explicating the link among annual solar cycles, geographic location, and eye growth regulation warrants further investigation.

## Supplementary Material

Supplement 1
