# Supplementary material for: Annual Solar Cycles: Investigating Seasonal Variations in Southern Norway and Their Potential Role in Human Eye Growth
Source: Invest Ophthalmol Vis Sci. 2026 Aug 21;67(10):54. doi: 10.1167/iovs.67.10.54 (PMC13505796; doi:10.1167/iovs.67.10.54)

Supplemenatary Material

Annual solar cycles: Investigating seasonal variations in Southern Norway and their potential role in human eye growth

Rigmor C. Baraas[*,1], Stuart J. Gilson[1]

[1]National Centre for Optics, Vision and Eye Care, University of South-Eastern Norway, Kongsberg, Norway.

[*]Corresponding author: rigmor.baraas@usn.no

Table S1 Cumulative alpha-opic EDI ratios based on estimated marginal means (EMMs), 95% confidence intervals (CIs), and Tukey-adjusted p-values for pairwise comparisons (Summer is reference).

		S:L ratio		MEL:L ratio		S+MEL:L+M ratio		L:M ratio	
		Mean	95% CI	Mean	95% CI	Mean	95% CI	Mean	95% CI
Summer	Morning	0.93	0.86–1.00	0.96	0.89–1.03	0.95	0.88–1.02	1.01	0.94–1.08
	Midday	0.92	0.86–0.99	0.95	0.89–1.02	0.94	0.88–1.02	1.01	0.94–1.09
	Afternoon	0.95	0.89–1.02	0.98	0.91–1.05	0.97	0.90–1.04	1.01	0.94–1.08
Autumn	Morning	0.98	0.91–1.05	0.99	0.93–1.07	0.99	0.92–1.06	1.01	0.94–1.08
	Midday	0.92	0.86–0.99	0.95	0.89–1.02	0.94	0.88–1.01	1.02	0.95–1.09
	Afternoon	1.05	0.98–1.13	1.04	0.97–1.12	1.04	0.97–1.12	0.99	0.92–1.06
Winter	Morning	1.36***	1.27–1.46	1.24***	1.16–1.34	1.27***	1.18–1.36	0.94	0.88–1.01
	Midday	1.22***	1.14–1.32	1.15**	1.07–1.24	1.17***	1.09–1.25	0.96	0.90–1.03
Spring	Morning	1.07	1.00–1.15	1.05	0.98–1.13	1.06	0.98–1.13	0.99	0.92–1.06
	Midday	1.02	0.95–1.10	1.02	0.95–1.10	1.02	0.95–1.10	1.00	0.93–1.07
	Afternoon	1.18**	1.09–1.26	1.12*	1.04–1.20	1.13*	1.05–1.22	0.97	0.91–1.04

p-values <0.05*, <0.01**, <0.001***

Figure S1: Cumulative alpha-opic EDI during morning and midday outdoor recess, as well as late afternoon outdoor activities for S-, MEL- and L-opsin. Measurements were averaged over 9-day windows centred on each equinox/solstice date, based on 360–830 nm wavelengths and calculated for a 10-year-old child. Summer is shown on the left as this is the reference in the statistical analysis.

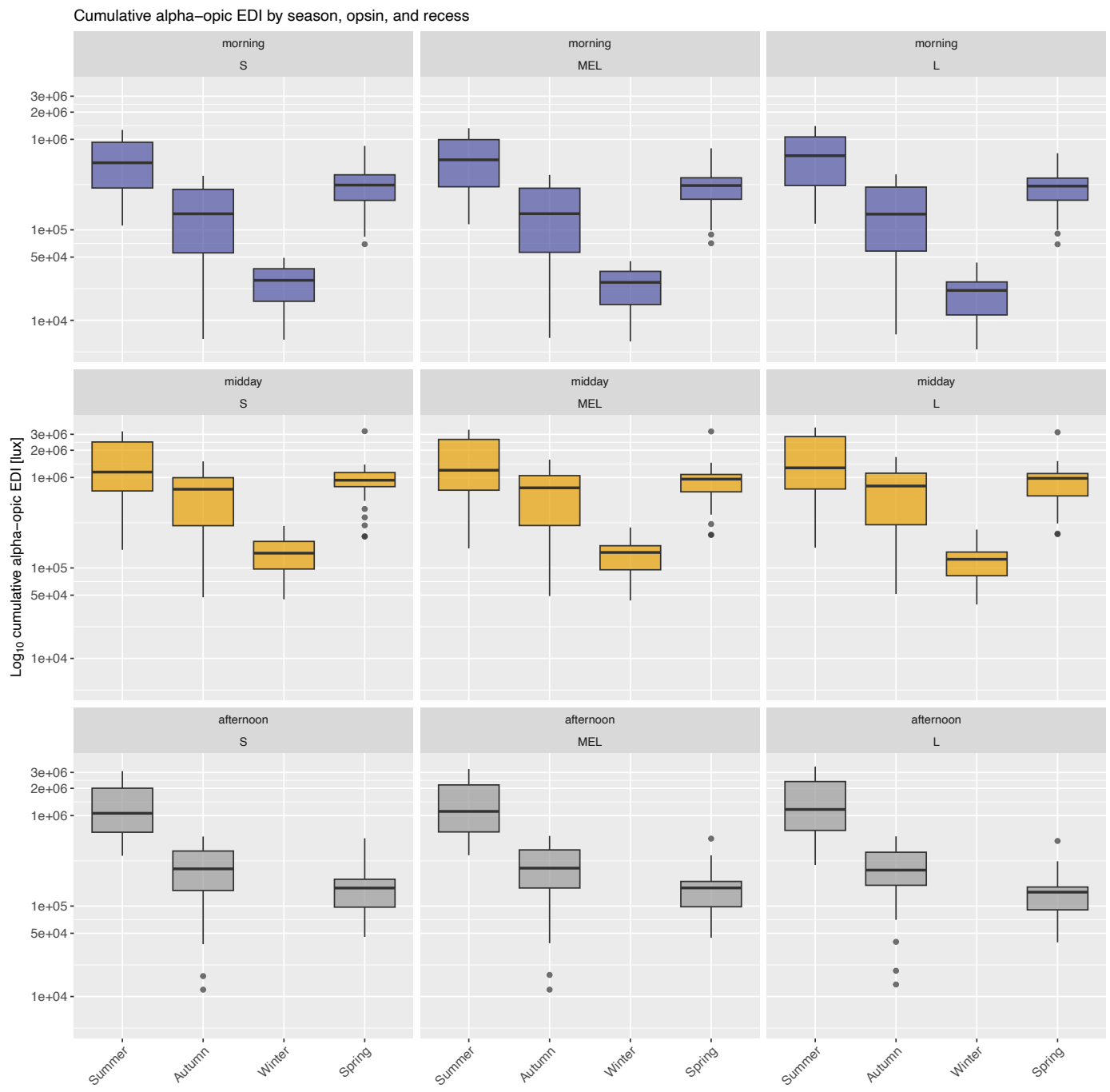

Figure S2: Cumulative available alpha-opic EDI ratios, during morning and midday outdoor recess, as well as late afternoon outdoor activities for S:L, MEL:L, L:M and (S+MEL):(L+M) opsin ratios. Measurements were averaged over 9-day windows centred on each equinox/solstice date, based on 360–830 nm wavelengths and calculated for a 10-year-old child. Summer is shown on the left as this is the reference in the statistical analysis.

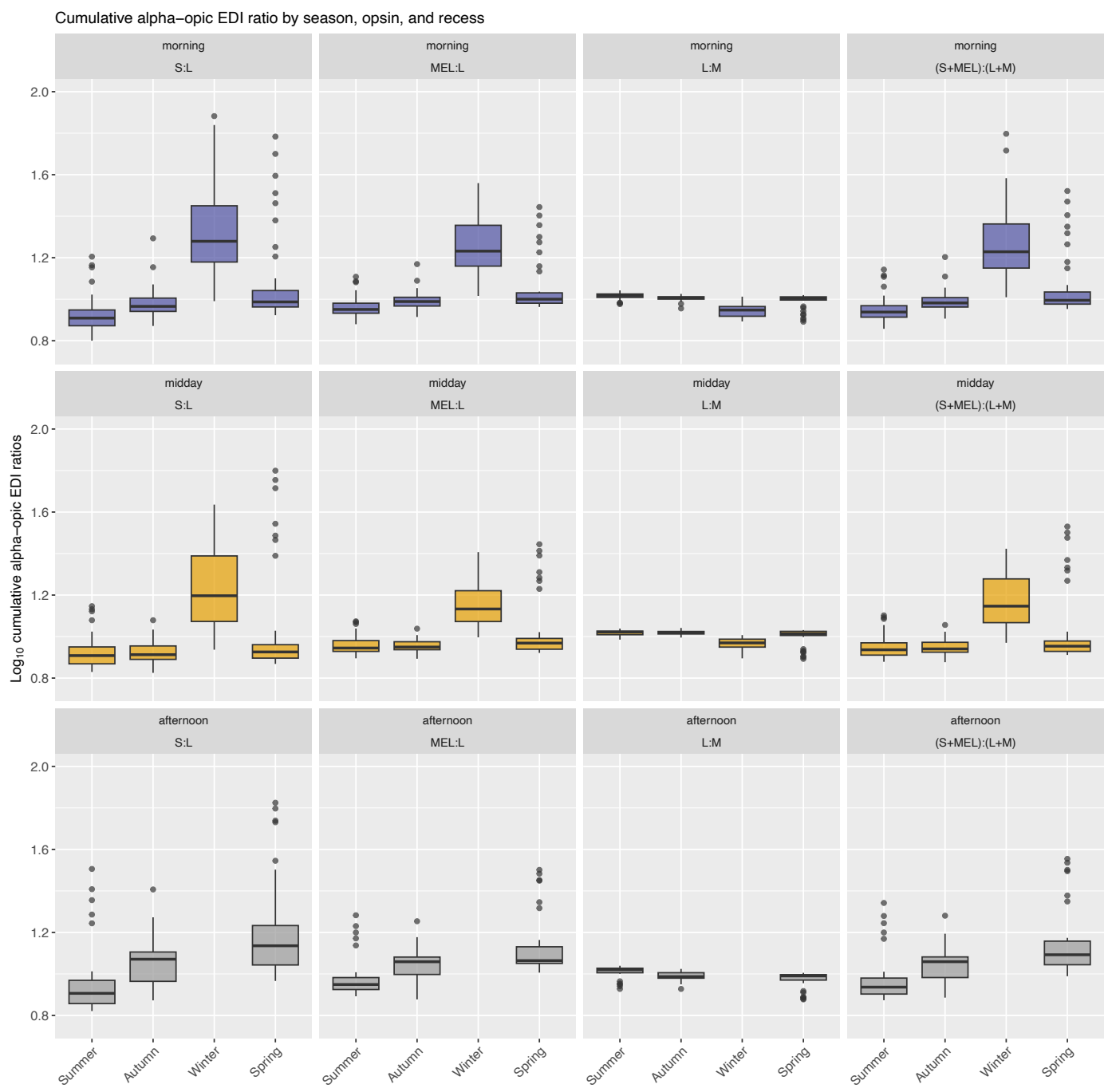

Supplement: Supplementary file 1 [file iovs-67-10-54_s001.pdf]
